# Long term respiratory morbidity in patients with vascular rings: a review

**DOI:** 10.1186/s13052-023-01430-x

**Published:** 2023-02-17

**Authors:** Federica Porcaro, Paolo Ciliberti, Francesca Petreschi, Aurelio Secinaro, Annalisa Allegorico, Antonella Coretti, Renato Cutrera

**Affiliations:** 1 Pulmonology and Cystic Fibrosis Unit, Bambino Gesù Children’s Hospital, IRCCS, Rome, Italy; 2 Cardiology Unit, Bambino Gesù Children’s Hospital, IRCCS, Rome, Italy; 3 Advanced Cardiothoracic Imaging Unit, Bambino Gesù Children’s Hospital, IRCCS, Rome, Italy

**Keywords:** Complete vascular rings, Incomplete vascular ring, Airway compression, Tracheomalacia, Children

## Abstract

Abnormalities in position and/or branching of the aortic arch can lead to vascular rings that may cause narrowing of the tracheal lumen due to external compression, or constriction of the oesophagus, causing symptoms that vary in relation to the anatomical vascular pattern and the relationship between these structures. Respiratory morbidity related to external airways compression is a major concern in children affected by vascular rings. Clinical presentation depends on the severity of the tracheal lumen reduction and the presence of associated tracheomalacia. Recurrent respiratory infections, wheezing, atelectasis, and hyperinflation are mostly reported. As they are nonspecific and therefore difficult to recognize, attention should be given to all children with history of respiratory distress, extubation failure, noisy breathing, and recurrent respiratory infections. Early diagnosis and referral to specialized centres can prevent the long-term complications and improve the respiratory outcomes of these patients.

## Introduction

Abnormalities in position and/ or branching of the aortic arch can lead to a complete or incomplete vascular ring (VR) that encircles and compresses the trachea, the bronchi and/or the oesophagus. Vascular rings (VRs) account for < 1% of congenital cardiac defects [1, 2]. Their real incidence is difficult to establish as the clinical presentations are non-specific and range from early onset of symptoms to asymptomatic clinical setting into adulthood [3, 4]. Though most VRs are isolated, congenital heart defects and chromosomal abnormalities can be associated [5, 6]. Extrinsic airway compression (EAC) and malacia are common complications of vascular rings. Otherwise, tracheal stenosis due to complete cartilaginous rings is found in association with left pulmonary artery sling (LPA sling).

Apparent life-threatening events (ALTE), apnoea, respiratory distress during airway infection and need of intubation, recurrent respiratory infections, barky cough, stridor, wheezing, and dyspnoea on exertion are all symptoms affecting patients with airway compression, associated malacia and tracheal stenosis due to VR [7]. Additional symptoms such as failure to thrive and dysphagia are reported in patients with oesophagus compression [8]. When prenatal diagnosis is not available and reported symptoms are suspicious for EAC by VR, a complete diagnostic workup should be considered by clinicians [9].

### Vascular rings

Due to the strong relationship of the thoracic vascular structures with the trachea and bronchi, any anatomic variation leading to complete or incomplete VR [Table 1] may produce several grade of EAC that appears typically pulsatile and located in characteristic positions [10].

**Table 1:** Type of vascular abnormalities of the aortic arch leading to airway compression

**Complete vascular rings**
Double aortic arch
Right aortic arch with mirror image branching and left ligamentum arteriosum
Right aortic arch with aberrant left subclavian artery and left ligamentum arteriosum
Circumflex aorta
Left aortic arch with aberrant right subclavian artery and right ligamentum arteriosum
**Incomplete vascular rings**
Right aortic arch with aberrant left subclavian artery and right ligamentum arteriosum
Left aortic arch with aberrant right subclavian artery
**Other common vascular abnormalities causing airway compression**
Left pulmonary artery sling
Innominate artery compression syndrome

As reported by recent studies, right aortic arch (RAA) with aberrant left subclavian artery (ALSA) is the most common complete VR [7, 11–13], followed by double aortic arch (DAA) [7, 14]. Innominate artery (IA) compression accounts 3 to 20% of cases of incomplete vascular ring, followed by left pulmonary artery (LPA) sling [15]. Less common complete VR subtypes are: RAA with mirror-image branching (MB), and retro-oesophageal course of the left- sided arterial duct between the right-sided descending aorta and the left pulmonary artery; circumflex retro-oesophageal aortic arch, and left aortic arch (LAA) with aberrant right subclavian artery (rSa) when the arterial duct is right-sided (RLA) [16].

**Fig. 1:**
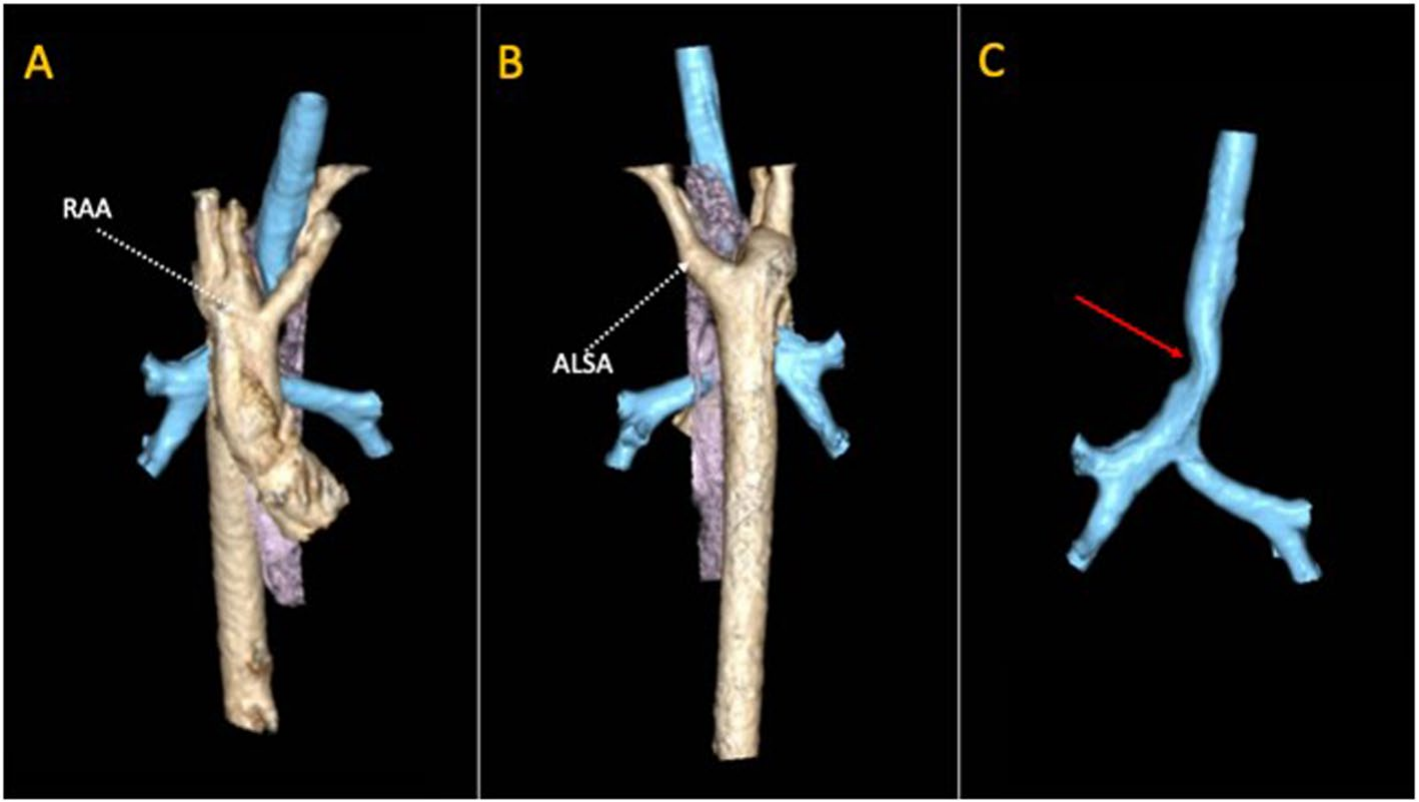
3D volume rendering reconstruction of a cardiac CT in patient with Right Aortic Arch (RAA) and Aberrant Left Subclavian Artery (ALSA). The vascular structures together with the left duct ligament (not visible on CT) completely encircle the trachea (in blue) and the oesophagus (in violet). Panel **A**: front view. Panel **B**: back view. Panel **C**: trachea with the narrowing (red arrow) at the level of the ring

Among all patients with RAA, a total of 65% of them have a true vascular ring due to the presence of a retroesophageal left subclavian artery with a left ligamentum arteriosum (LLA) [Fig. 1] [17]. The aneurysmal dilatation at the base of the left subclavian artery is known as Kommerell diverticulum (KD), that can be an independent cause of airway compression when enlarged > 1.5 times the diameter of the subclavian artery [18, 19]. The division of the ligamentum arteriosum allows to free the trachea from the ring, giving relief of airway compression and promoting the normal development of tracheal cartilage. When dissection of KD is necessary, translocation of the aberrant left subclavian artery is also performed [19].

**Fig. 2:**
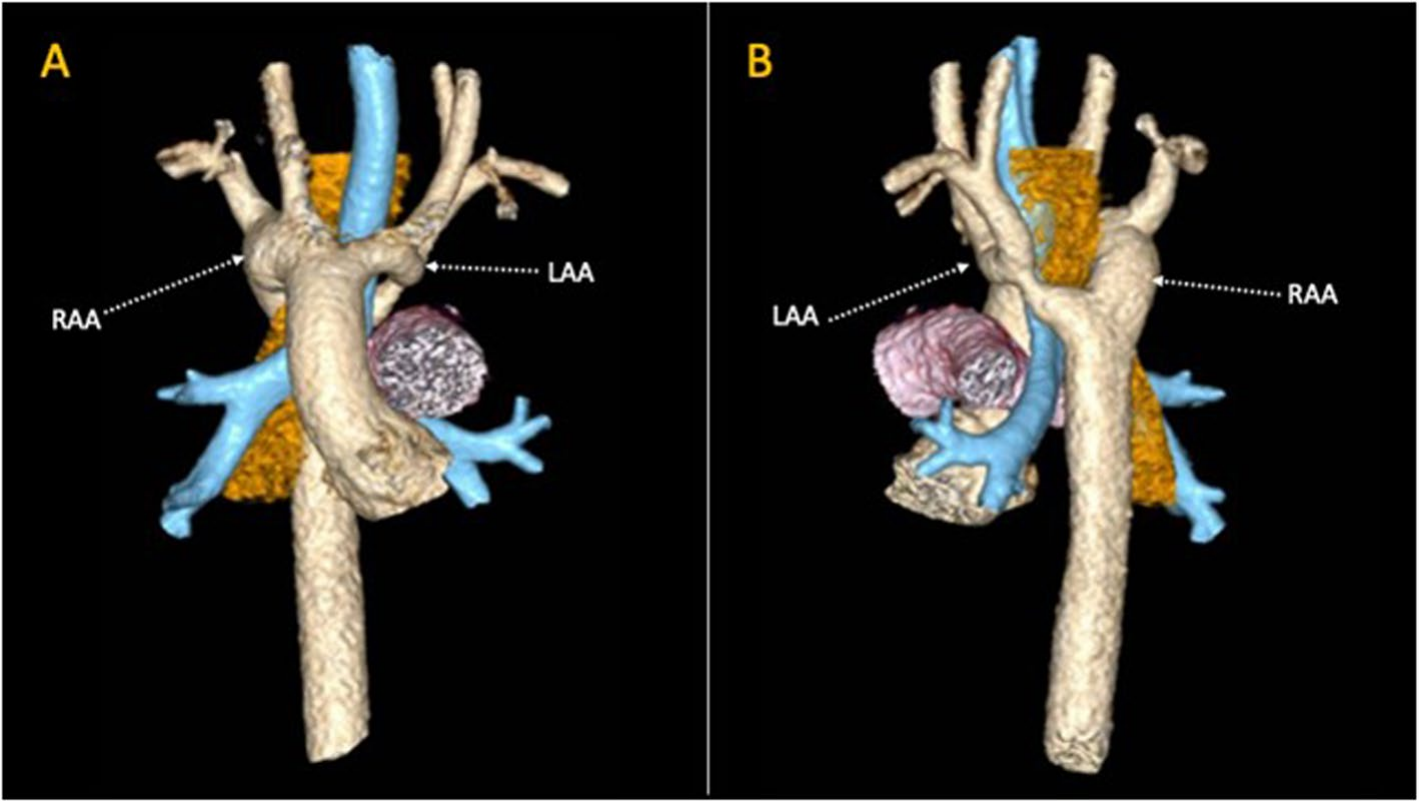
3D volume rendering reconstruction of a cardiac CT in patient with Double Aortic Arch, constituted by a dominant right aortic arch (RAA) and a diminutive Left Aortic Arch (LAA). The vascular structures together completely encircle the trachea (in blue) and the oesophagus (in orange). Pulmonary artery in violet. Panel **A**: front view. Panel **B**: back view

DAA [Fig. 2] with dominant right arch (80%), or dominant left arch (10%), or balanced arches (10%) [17] is a true vascular ring that accounts about 30 to 45% of all VRs [20]. The smaller of the two arches, or the atretic arch, should be divided [21].

**Fig. 3:**
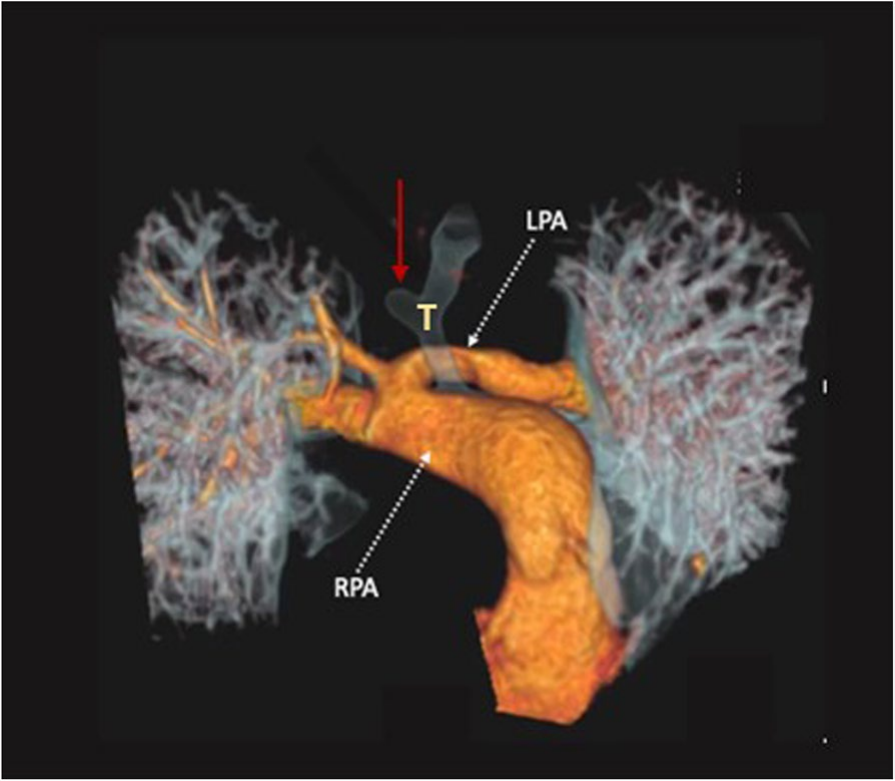
3D volume rendering reconstruction of a cardiac CT in patient with Left Pulmonary arterial sling. The Left Pulmonary Artery (LPA) arises posteriorly and distally from the Right Pulmonary Artery (RPA), coursing posteriorly to the trachea (T). A tracheal bronchus (commonly associated to LPA sling) is also detected (red arrow)

LPA sling is a rare vascular anomaly where the LPA does not arise from the pulmonary trunk but takes its origin distally from the posterior aspect of the right pulmonary artery [Fig. 3**]**. The LPA then makes a turn to the left, and courses toward the left lung passing between the lower trachea and oesophagus. Therefore, a vascular sling is formed around the right side of the trachea. The arterial duct, or its ligament, connects the pulmonary trunk to the descending aorta on the left side of the trachea, completing the vascular ring. The sling is almost always associated with an airway anomaly.

LPA sling sometimes can lead to right lung hyperinflation [17]. Tracheal stenosis due to complete cartilaginous rings is detected in 75% of patients affected by LPA sling [3] and is often responsible of the poor prognosis of this vascular abnormality. Surgical repair with LPA reimplantation and slide tracheoplasty is required and the mortality and morbidity rate are directly related to the extent of the tracheal stenosis [3].

The innominate artery compression syndrome cannot be considered a true vascular ring because the trachea is crossed and compressed anteriorly (from the left to the right) by the IA rising abnormally from the aorta on the left side of midline and coursing rightward in front of the trachea [3]. The suspension of IA is indicated when there is the evidence of more than 80% compression of the tracheal lumen by bronchoscopy [3].

The mere presence of a vascular ring is not an indication in itself for surgical intervention. It is important to know that symptoms in small children may disappear as the child and chest cage grow. It is generally accepted, however, that surgical intervention should be undertaken if the patient is symptomatic, and or in presence of significant compromise to the airways. Unnecessary delay may cause further tracheobronchial damage, leading to chronic compromise of the airways, with poor outcome in term of symptoms relief even after the correction [3, 5, 16].

Some Authors advocate that the decision for surgery should be based on the severity of symptoms and not just the degree of airway compression [22]. Indeed, Myer and colleagues [23] included reflex apnoea, failure of medical management of severe respiratory distress after 48 h, and prolonged intubation among absolute criteria. At a later time, recurrent tracheobronchitis or bronchopneumonia, exercise intolerance, dysphagia, or failure to thrive with associated subglottic stenosis, asthma, cystic fibrosis, or prior tracheoesophageal fistula repair were considered relative criteria [10].

Very recently, Said and Biermann confirmed in two different papers the indication to treat surgically all complete VRs (DAA, RAA with LLA, LAA with RLA) and LPA sling, reserving surgery to symptomatic incomplete VRs or those without symptoms and associated cardiac defects [12, 19].

### Respiratory symptoms

Most of the patients with complete VRs and LPA sling have respiratory symptoms that often occur early in life (1 – 6 months), becoming progressive with the growth of the intrathoracic structures. However, significant tracheal compression may be present in infants even without symptoms [3, 24].

Clinical manifestations vary with the severity of encroachment on the trachea, bronchus or oesophagus by the abnormal vascular structures. The severity of compression not only depends on the type of anomaly, but is also related to the size and shape of the thoracic cage enclosing the vascular structures and airway [16].

**Fig. 4:**
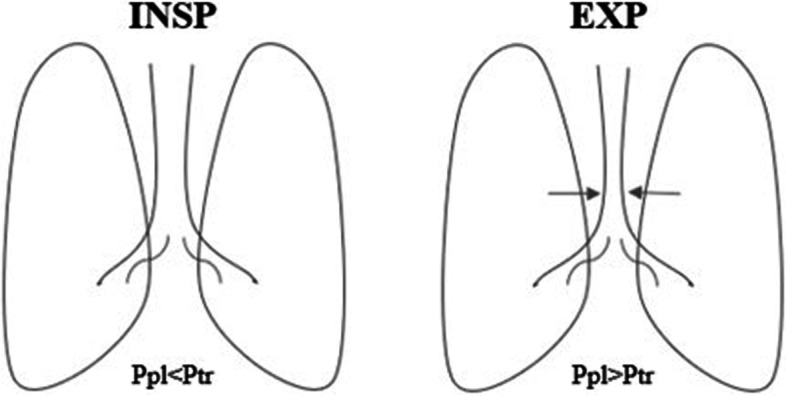
Effect of intrathoracic pressure (Ppl) variation on tracheal lumen during a) inspiration and b) expiration phases. Ppl overcomes intra-tracheal pressure (Ptr) during expiration, leading to the lumen collapse (black arrows)

Symptoms vary from apnoea and cyanosis to stridor, barky cough, wheezing, shortness of breath and dysphagia especially for solid food [2, 3, 7, 10, 12, 25]*.* In addition, because the tracheal compression impairs the muco-ciliary clearance leading to the stagnation of secretions, patients and their parents report history of chronic cough poor response to medical treatment, recurrent bronchopneumonia, fatigue during physical exertion and need of invasive respiratory support during respiratory exacerbation in the most severe cases [26]. Reported symptoms usually become evident during expiration, when the intrathoracic pressure (Ppl) overcomes the intratracheal pressure (Ptr), leading to the worsening of airway narrowing [10]. Symptoms’ worsening is explained by Bernoulli’s principle, according to which the increase in the speed of a fluid occurs simultaneously with a decrease in pressure in the point where the channel narrows [Fig. 4].

Because the described clinical picture is often misinterpreted as laryngomalacia, bronchiolitis, asthma, and gastroesophageal reflux, clinician should keep in mind the possibility of a VR in children with recurrent respiratory symptoms with or without digestive symptoms and poor response to medical treatment [27–30].

### Respiratory functional assessment

**Fig. 5:**
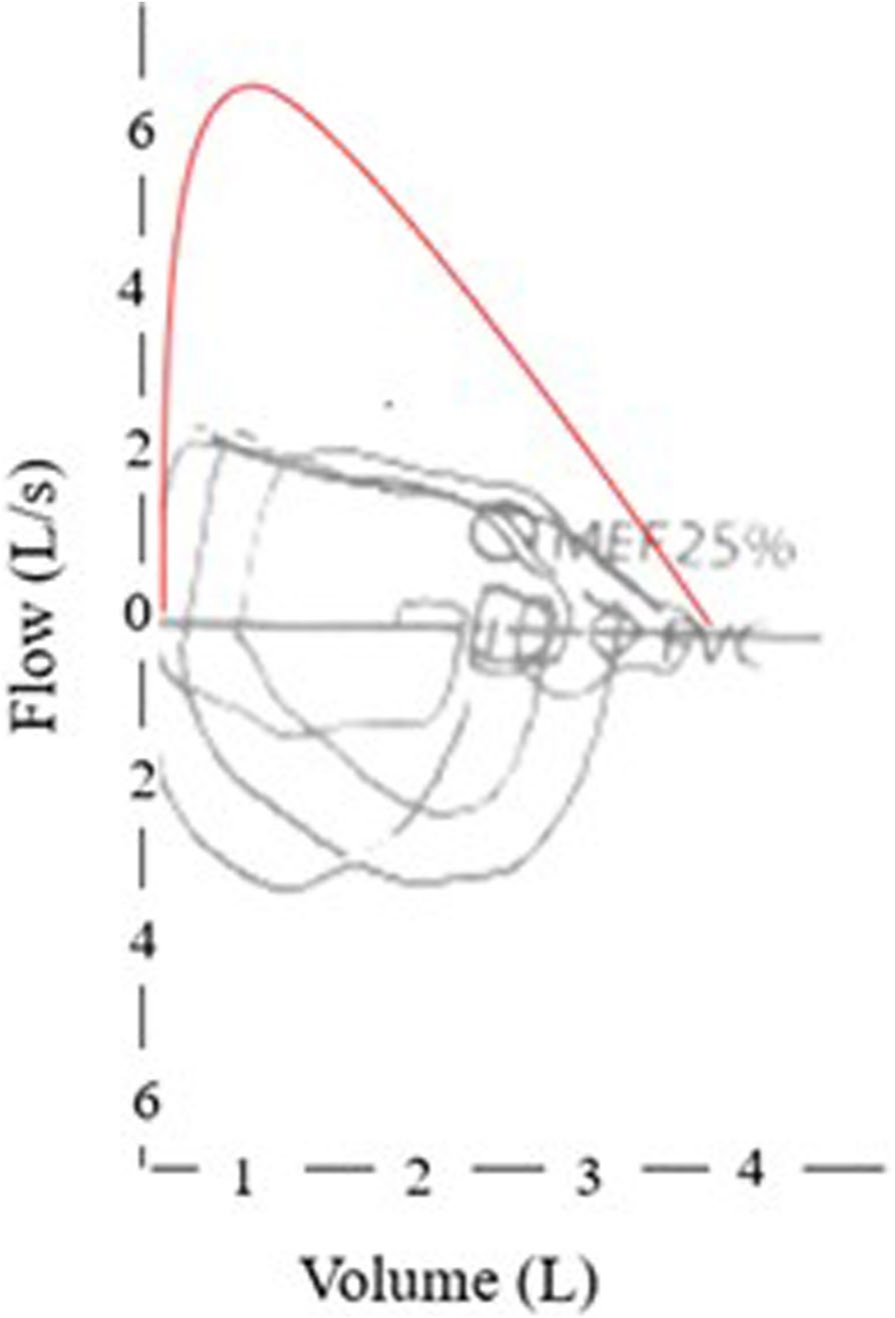
Maximal expiratory flow-volume curve in a patient with complete vascular ring (RAA with mirror image branching and LLA). Note the flattening of the proximal portion of the expiratory flow-curve due to the external compression/tracheomalacia that affects to expiratory flow rates in the large airways but not in the peripheral ones. FEV1 2.12 L (85%), FVC 3.00 L (102%), FEV1/FVC 83%, FEF25-75% 1.82 L/s (65%)

In addition to the clinical history, spirometry should be considered as non-invasive, fast, and available easily test in children aged over 6 years. It can help the physician to support the initial suspicion showing the abnormality of the flow-volume curve shape. Indeed, the diminished expiratory flow in the initial phase followed by an upward deflection and then the continuation of exhalation on the flow-volume curve is indicative of intra-thoracic obstruction [Fig. 5]. The notch, not variating after inhaler short bronchodilator administration, reflects the sudden diminution of flow at the beginning of expiration when the compressed airway collapses [31].

Because spirometry provides a static measure of lung function, it doesn’t evaluate the respiratory abilities to perform exercise, which is considered very important in the psychological and physical development of children and adolescents. Therefore, the exercise challenge test is helpful to reproduce exercise induced symptoms reported by patient [32], giving a complete idea of the clinical situation. The test should be obviously suitable for the patient’s age (step test, six-minutes walking test, exercise test on treadmill or cycle ergometer). Because it can help to identify and quantify the limitations during exercise, the derived information can be helpful for the therapeutic choice which may include a conservative or not attitude [33]. Moreover, clinician may use both static and dynamic lung function tests during the post-operative period to monitor the obstructive pattern and detect the resolution of airflow limitation.

### Diagnostic assessment of airway compression/collapse

VR can be accurately diagnosed by fetal ultrasound improving prognosis in these patients, as it enables proper timing of additional postnatal investigation and surgery [11]. Chest x-ray is not routinely recommended to diagnose VR, even though it can be useful to show the location of the aortic arch in relation to the trachea [34]. Both diagnostic tests are not able to study the impact of the vascular abnormality on airways, therefore additional investigations should be considered.

Computed tomography with angiography (CTA) is an important tool that has currently replaced barium contrast esophagogram as it allows a careful simultaneous assessment of vascular abnormalities and airway involvement [35]. In addition, dynamic image analysis during a complete respiratory cycle (inspiration and expiration) provides a fully evaluation of the airway collapse that shows a high correlation with the degree of respiratory impairment [36, 37]. Endoluminal volume rendering also allows virtual tracheobronchoscopic demonstration of the airway.

CTA is most widely used compared to magnetic resonance imaging (MRI) because of its fast scanning time, enabling to perform the investigation without sedation/anesthesia even in very small and uncooperative children, and the ability to thoroughly assess the airway [38]. Nevertheless indication to CTA investigation must be carefully assessed, valuing risk/benefit ratio, since radiation exposure remains a major concern [38]. Flexible laringotracheo-bronchoscopy performed under light sedation and spontaneous breathing allows the dynamic evaluation of the tracheobronchial tree, revealing the localization, extension and the estimation of the airway malacia severity [10]. This exam is often the first investigation performed in children effected by persistent stridor or recurrent respiratory symptoms despite medical treatment. The finding of a pulsatile narrowing raises the suspicion of vascular compression and requires further examinations defining the underlying vascular abnormality and the degree of airway compression. The spatial location of compression detected on flexible bronchoscopy can suggest the type of VR that in any case must be confirmed with imaging: right aortic arc (RAA) usually compresses the right posterior distal trachea; double aortic arch (DAA) usually compresses the right anterior and posterior distal trachea; LPA sling usually causes compression of the right anterior distal trachea and right mainstem bronchus as well as the posterior trachea; innominate artery compression usually presents as anterior mid-tracheal compression [10]. Differently, once the diagnosis has already been made, airway endoscopy represents a complementary exam to be performed during correction to verify the efficacy of surgical manoeuvre [39].

Unfortunately, there are no guidelines on the better diagnostic work up for children suspected of VR. However, it’s reasonable that children with history of recurrent respiratory symptoms poor response to medical treatment, symptoms and physical examination of airway obstruction, dysphagia, and failure to thrive should undergo to flexible laryngo-tracheo-bronchoscopy to assess for laryngomalacia, vocal fold mobility, tracheal stenosis or malacia [40]. The compression or collapse of 33% or less of the tracheal length is considered suggestive of extrinsic compression, while intrinsic tracheomalacia is considered when more of the trachea (> 33%) is involved [10]. In addition, the detection of compression of the right anterior and posterior tracheal walls or mainstem bronchus is considered indicative of complete VRs or pulmonary artery sling for which other imaging investigations are required. The grade of tracheal lumen reduction on flexible bronchoscopy (FBs) is another factor to consider when choosing whether to perform additional investigations. Indeed, the patient should undergo a chest angio-CT or MRI when anterior or posterior tracheal compression greater than 50% is found. When less than 50% of tracheal collapse id observed, the decision to make CTA or MRI to avoid misdiagnosis should be based on reported symptoms [10]*.*

### Long term respiratory outcomes

Morbidity and mortality in patients after surgical correction have been reported to a greater extent, in those with respiratory distress at presentation, underlying genetic condition, associated congenital tracheal stenosis or severe bronchomalacia, tetralogy of Fallot with absent pulmonary valve syndrome, underlying complex cyanotic heart disease and need of early respiratory support [7].

Since obstructive respiratory symptoms may persist for weeks or months after surgical intervention, a careful respiratory evaluation is required both in the short and long-term period. Anyway, it should be considered that symptoms’ persistence depends on timing and technique of surgical correction as well as the presence of residual malacia.

Studies describing respiratory symptoms trend after surgery correction are quite scarce, and reported follow up period ranges between six months and eleven years [2, 12, 14, 25, 26, 41–49]. A review of scientific literature was carried out using PubMed. The search was limited to the paediatric age. The search terms “respiratory outcomes” and “vascular rings” were used, and only English language articles were selected. The search resulted in 43 articles from 1992 to present; all titles and abstracts were reviewed. Only fourteen met our goal of assessing the course of symptoms after treatment [Table 2].

**Table 2:** Papers reporting long term respiratory outcomes in symptomatic children affected by VRs

Authors	Type of corrected vascular anomaly	Cardiac comorbidities	Other comorbidities	Median age at the time of surgery	Follow up	N. of available symptomatic patients to follow-up	% of population with improved symptoms
Chun, 1992 [41]	RAA + ALSA, DAA	*N.A*	*N.A*	7 mo	12.5 mo	32	84%
Bonnard, 2003 [2]	DAA, RAA + ALSA, RAA + LDA	VSD, ASD, persistent PDA	Di George, Rubinstein-Taybi syndrome EA, lobar emphysema	*N.A*	37.4 mo	60	68%
Shanmugam, 2005 [42]	DAA	VSD, PDA, CoA	Down, Di George, Pader Willi syndromes, left lower lobe sequestration with renal and sacral agenesis and anal atresia, TEF, sleep apnea, Congenital hip dysplasia, HT, Spina bifida with dysmorphism	5.7 mo	2 y	28	75.9%
Turner, 2005 [43]	RAA + LLA, DAA, LPA, RAA + ALSA	*N.A*	Down, Di George, syndromes, anal atresia, tracheal stenosis, congenital lobar emphysema	*N.A*	18 mo	20	90%
Alsenaidi, 2006 [44]	DAA	*N.A*	*N.A*	6 mo	6.5 mo	74	66%
Ruzmetov, 2009 [14]	DAA, RAA + LLA, rSa, LPA, IA	VSD, ASD, IAA, AS, TOF, SV, CoA, DTS, DORV, TA, MVS	Di George, Down and Goldenhar syndromes, Dandy-Walker cyst syndrome, hydrocephalus, cleft lip-palate, TEF, distal tracheal stenosis, unperforated anus, BPD	5 mo	1 y	135	75%
Herrin, 2017 [45]	DAA, RRA + ALSA	*N.A*	*N.A*	1.8 y	1 mo	125	74%
Naimo,2017 [46]	DAA, RAA + ALSA, RAA + MB + LLA, LAA + RLA	VSD, ASD, TOF, DORV, PDA	*N.A*	1 y	11.4 y	121	86%
Franҫois, 2017 [47]	DAA, RAA + ALSA, LAA + RLA, LPA	VSD, ASD, TOF, DORV	Di George, CHARGE, Down syndromes, VCF, EA	1 y	6 mo	55	83%
Schmidt, 2018 [25]	DAA, RAA + ALSA, RAA + LLA	VSD, ASD, PDA, TVi, MVi	Non-malignant neurofibromato, CANA1S gene mutation, CHARGE syndrome, laryngo-/tracheo-/bronchomalacia, asthma, ADHD, MBL mutation, SH purpura, choanal atresia	1.4 y	6.8 y	21	14%
Depypere, 2019 [48]	DAA, RAA + ALSA, RAA + MB	$	$	8 mo	10 y	36	72%
Callahan, 2020 [49]	DAA, RAA + ALSA, RAA + MB	*N.A*	DiGeorge, Trisomy 21, CHARGE, PHACE, Rosai-Dorman syndrome other mutation, deletion, duplication	9.9 mo	17.4 mo 10.9 mo	34 25	65% (aberrant subclavian artery) 43.5% (DAA)
Said, 2021 [12]	DAA, RAA + ALSA, LAA + rSA, LPA	VSD, ASD, TOF, CoA, TA, PVS	DiGeorge, Noonan, Klippel-Feil syndrome, craniocynostosis, myotonic dystrophy type 1, CHARGE syndrome, EA + TEF, polysplenia with absent distal pancreas, HLH	7.3 mo	3 ± 5 y	42	93%
Yu, 2022 [26]	DAA, RAA + LLA, LAA + RLA, LAA + rSa, LPA, IA	VSD, ASD, TOF	Di George, Down and Goldenhar syndromes, imperforate anus, unilateral renal agenesis, cleft palate, polydactylism, hypospadias	10 mo	4.3 ± 2.9 y	N.A	94.6%

Legend: *AS* Aortic stenosis, *ASD* Atrial septal defect, *ADHD* Attention deficit hyperactivity disorder, *BDP* Bronchopulmonary dysplasia, *CANA1S gene* Calcineurin A1 gene, *CoA* Coarctation of the aorta, *DTS* Distal tracheal stenosis, *DORV* Double-outlet right ventricle, *EA* Esophageal atresia, *HLH* Familial hemophagocytic lymphohistiocytosis, *HT* Hashimoto’s thyroiditis, *IAA* Interrupted aortic arch, *MBL* Mannose binding lectin deficiency, *MVi* Mitral valve insufficiency, *MVS* Mitral valve stenosis, *PVS* Pulmonary valve stenosis, *SH* Schönlein- Henoch’s purpura, *SV* Single ventricle, *VCF* Velocardiofacial syndrome, *VSD* Ventricular septal defect, *TOF* Tetralogy of Fallot, *TEF* Tracheoesophageal fistula, *TGA* Transposition of the great arteries, *TVi* Tricuspidal valve insufficiency, *TA* Truncus arteriosus, *UVH* Univentricular heart. *N.A* Not available, *Mo* Months, Y: years

^$^ The presence of comorbidities has been considered as exclusion criteria

Chun and colleagues evaluated the outcomes of 37 survivors affected by VRs. None of these patients required prolonged ventilatory support or tracheostomy in the short term. Respiratory arrest, pneumonia and pneumothorax were recorded among respiratory complications immediately after surgery. Median length of follow-up of survivors was 12.5 months. Five patients were lost in the follow up; 12/32 were asymptomatic and 15/32 had mild respiratory symptoms at 6 months to 1 year after repair [41].

Bonnard et al. described their experience on 62 children affected by VRs. Authors reported a complete improvement in 68%, a partial improvement in 17%, and no improvement in 15% of cases after intervention, followed up for a mean period of 37.4 months (range 12 to 159 months) [2].

Respiratory sequelae were also retrospectively examined by two different groups of researchers belonging to the same institution. Despite a small difference in the sample size, Authors reported a good trend of respiratory symptoms during a period of nearly 2 years with symptoms resolution in more than 70% of patients [42, 43].

Alsenaidi and coll. noted a high persistence of respiratory symptoms in their cohort of 81 DAA affected children undergone surgical correction. 34% of symptomatic patients had residual symptoms at a median follow-up of 6.5 months [44].

Ruzmetov reported his long term follow up (38-years) after surgical correction of 183 patients affected by aortic arch anomalies with tracheal compression. Postoperative complications occurred in 2% (3/180 of survivors) of treated patients and included tracheostomy because of severe distal tracheal compression and left true vocal cord paralysis. 75% of patients were free from compressive symptoms within 1 year from surgery [14].

Herrin et al. analysed 200 patients undergone surgery for VRs. Fifteen children (7.5%) experienced postoperative complications including tracheostomy, left vocal cord paralysis, pneumothorax and chylothorax. Only 125 patients were evaluated within 1 month from discharge, and 92 (74%) were asymptomatic from a respiratory point of view [45].

Naimo and coll. described 132 patients surgically treated for VRs, with a median follow up of 11.4 years. Overall 86% reported improvement of respiratory symptoms [46].

Franҫois et al. analysed the respiratory outcomes of 62 treated VRs who underwent to a mean follow-up period equal to 7.8 ± 5.8 years. Among the 55 symptomatic patients, freedom from residual symptoms has been recorded at 1 and 6 months in 63% and 83%, respectively [47].

Conversely, Schmidt and coll. reported a low percentage of respiratory improvement in their sample of 21 patients followed up for a median period of 6.8 years. Only 14% (3/21) of patients didn’t report any issue at the last follow up visit [25].

Partial symptoms’ improvement was observed at long term follow up (median time 8 years, IQR 5–12 years) of patients with isolated VR. Only 36/51 children completed the follow up at 10 years and 26/36 were free of symptoms at this moment [48].

Callahan et al. described the respiratory outcomes of 63 children affected by aberrant subclavian artery and double aortic arch undergone to surgery correction. Persistent breathing difficulties were recorded in 35% children with aberrant subclavian artery and in 56.5% with double aortic arch. The median time for the latest reported respiratory symptom was 17.4 months (IQR 3.0–27.5 months) and 10.9 months, respectively [49].

Also Said and coll. reported good respiratory outcomes for children affected by vascular ring/pulmonary artery sling. Vocal cord paralysis, pneumothorax, transient chylothorax and pneumonia have been reported after surgery correction in the short term period, while an overall clinical improvement has been described in 93% of symptomatic children following a mean follow up of 3 ± 5 years [12].

A very recent paper reported the long-term outcomes in children undergone vascular ring division and followed for a time of 4.3 ± 2.9 years: twenty patients (5.3% of the total population) had residual respiratory symptoms, the most frequent of which was stridor [26].

Globally these studies show a positive trend of resolution of symptoms after surgical correction. Nevertheless, the difference in percentage of symptom resolution, likely reflects discrepancy among the different cohorts in term of timing of intervention, anatomical variants, and prevalence of associated lesions.

**Fig. 6:**
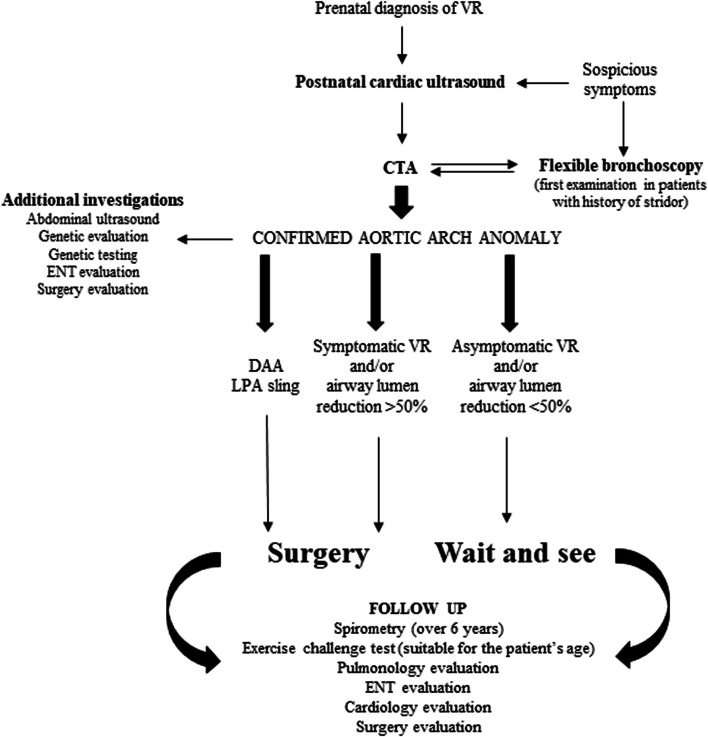
Algorithm for the diagnosis, treatment and monitoring of children affected by VR

Finally, based on the available literature about the treated topic and commented above, we designed an algorithm that reflects our daily practice. It includes the investigations required for the diagnosis, the indications for surgical treatment and the evaluations needed for monitoring the treated and non-treated patients during the follow up period [Fig. 6].

## Conclusion

Over the recent years there has been an increase in detection of VRs in the current era due to the increased rate of fetal diagnosis. Anyway, for patients with no fetal diagnosis, it should be considered that the clinical presentation is frequently non-specific and that a high index of suspicion is needed to allow diagnosis.

Indications for surgical repair are clear in the presence of symptoms; however, controversy exists about the indication for intervention and timing in asymptomatic patients. Generally, treatment is reserved for all symptomatic VRs and asymptomatic cases with DAA, marked Kommerell diverticulum, or in the presence of concomitant congenital heart disease where surgical repair is needed. Otherwise, conservative treatment might be reasonable in asymptomatic or mildly symptomatic cases.

Anyway, early diagnosis and surgery are imperative to reduce the long-term complications of tracheobronchial compression in children with symptoms or significant airway narrowing. Though postoperative morbidity and mortality are reported as low, respiratory symptoms’ resolution may require months to years from the surgery. Therefore, a multidisciplinary follow-up that includes a periodical pulmonology evaluation should be considered.

## Data Availability

Not applicable.

## Abbreviations

ALSA: Aberrant left subclavian artery
ALTE: Apparent life-threatening events
CTA: Computed tomography with angiography
DAA: Double aortic arch
EAC: Extrinsic airway compression
FBs: Flexible bronchoscopy
IA: Innominate artery
Ppl: Intrathoracic pressure
Ptr: Intratracheal pressure
LAA: Left aortic arch
LLA: Left ligamentum arteriosum
LPA sling: Left pulmonary artery sling
KD: Kommerell diverticulum
MRI: Magnetic resonance imaging
MB: Mirror branching
PDA: Patent ductus arteriosus
RAA: Right aortic arch
RLA: Right ligamentum arteriosum
rSa: Right subclavian artery
VR: Vascular ring
VRs: Vascular rings
